# Results of a client satisfaction questionnaire in a NHS psychotherapy department

**DOI:** 10.1192/bjo.2021.888

**Published:** 2021-06-18

**Authors:** Elizabeth Ogston, Kim Herbert, Lorraine McGuiness

**Affiliations:** NHS Lanarkshire

## Abstract

**Aims:**

This study aimed to assess the level of satisfaction patients feel towards their experience of attending for psychotherapy, in order to inform local management on the service being offered by the department.

**Background:**

This survey was conducted as part of routine service provision analysis by the psychotherapy department. It aimed to assess the level of satisfaction patients feel towards their experience of attending for psychotherapy, in order to inform local management on the service being offered by the department. Ethics committee confirmed this fulfilled “Service evaluation” criterion and the project was registered with the local NHS quality improvement register.

**Method:**

Patients who completed an episode of therapy were invited to complete a survey form. This consisted of a Client Satisfaction Questionnaire (CSQ-8) as well as four additional questions pertaining to patient satisfaction. The patient's therapist would inform administration staff of the patient's final appointment; administration staff would then issue the patient with a questionnaire which they were invited to complete and return in their own time. The questionnaires were completed anonymously and no reward was offered for completing the questionnaire. The therapies included group analysis, psychodynamic individual and Cognitive Analytic Therapy.

**Result:**

2 patients who had completed psychotherapy in 2018-2019 returned a completed questionnaire. The average and range responses were examined.

The average response was “4: Excellent” for the overall rating of the service received, and for 5 other questions on the CSQ-8 the average score was the highest possible. The average response was slightly lower on the question about whether the service met their needs “3: Mostly”, and on the question: Has the service you received helped you to deal more effectively with your problems? (3 yes, somewhat). The additional questions highlighted how important the setting and administration role played in the experience of therapy. The questionnaire also included a free text box giving the patient the opportunity to offer any other comments. Many of these included messages of gratitude and remarks on the impact therapy has had on their general wellbeing.

**Conclusion:**

In general it is encouraging to see that feedback provided through this survey was extremely positive. This was reflected both in the Likert scale questions and the free text box. Patients are described themselves as very satisfied with their experience within therapy and reflected a positive experience of the holding environment provided by the department as a whole.

(NO Funding received)

